# Supplementary material for: Mutational Analysis of Redβ Single Strand Annealing Protein: Roles of the 14 Lysine Residues in DNA Binding and Recombination In Vivo
Source: Int J Mol Sci. 2021 Jul 20;22(14):7758. doi: 10.3390/ijms22147758 (PMC8303780; doi:10.3390/ijms22147758)
Supplement: Supplementary file 1 [file ijms-22-07758-s001.zip › ijms-1281629-supplementary.pdf]

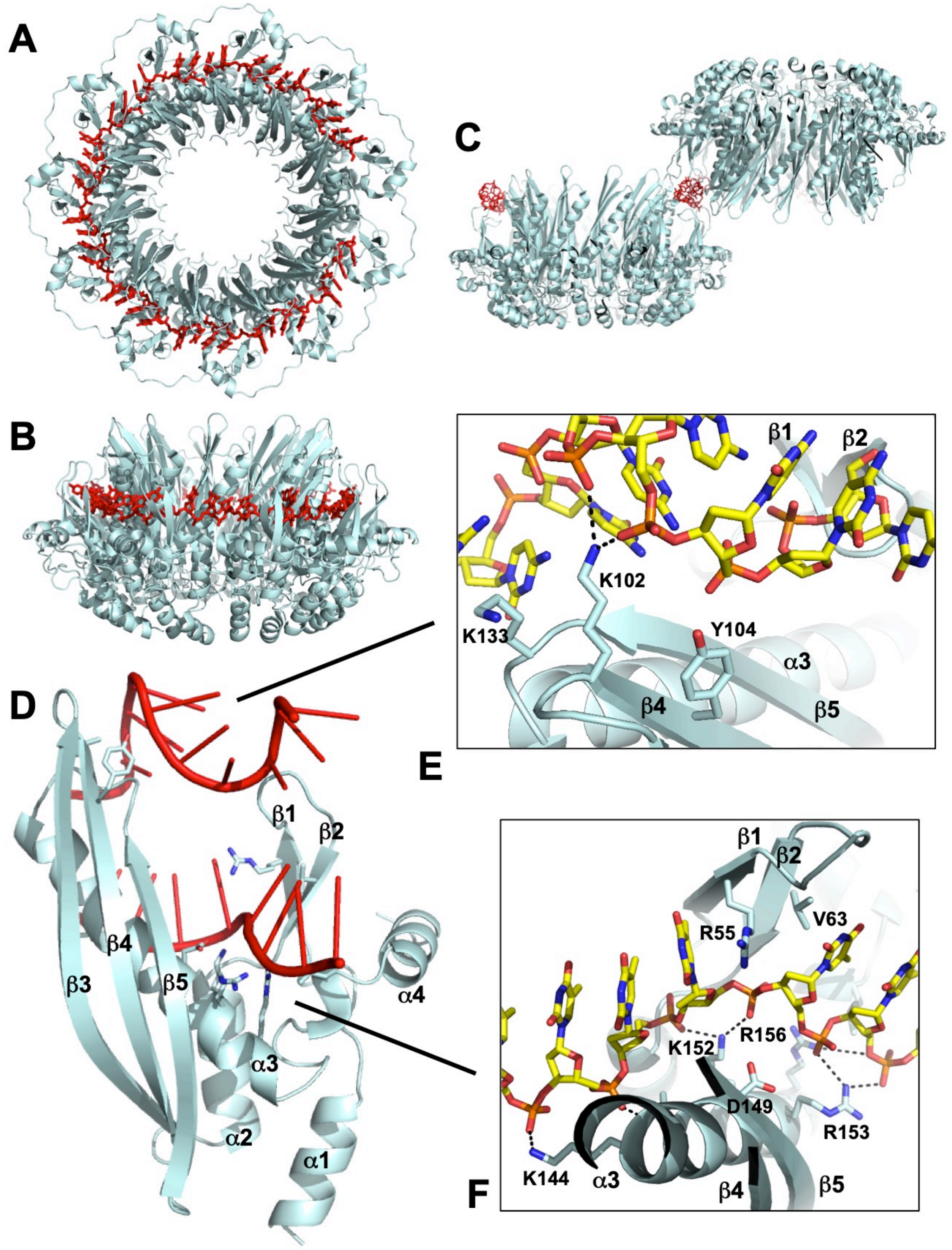

Figure S1. Crystal structures of the N-terminal DNA binding domain of human Rad52 in complex with ssDNA [1]. (**A, B**) Top and side views of a complex with dT40 ssDNA bound to the inner site, deep at the base of the DNA-binding groove (PDB code 5XRZ). (**C**) Side view of a structure with a shorter segment of ssDNA bound to the outer site at the upper rim of the groove, bridging two rings in the crystal lattice (PDB code 5XS0). (**D**) Composite structure of a monomer and its ssDNA from panels A and B, but with the ssDNA from panel C superimposed, to show the potential juxtaposition of strands. Notice that while the ssDNA bound to the inner site has its bases exposed and pointing up, the ssDNA bound to the outer site is in a right-handed helical conformation. (**E, F**) Close-up views of the interactions at the outer and inner sites, respectively. Notice that the interactions at the inner site are considerably more extensive.

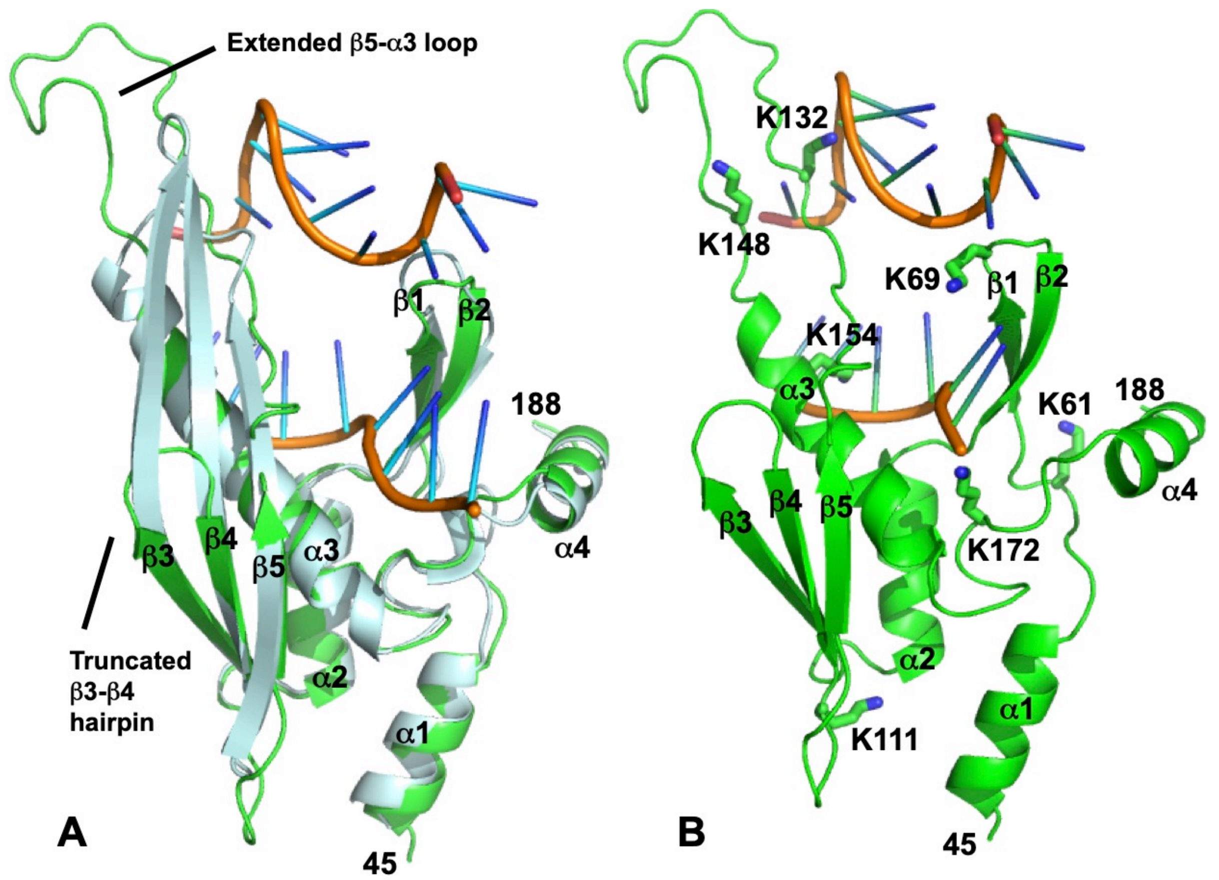


```
                                                     α1                         β1                β2        α2
                                                HHHHHHHHHHHH                 EEEEEE            EEEEE  HHHHHHHHHHH

Rad52   1 MSGTEEAILGGRDSHPAAGGGSVLCFGQCQYTAEEYQAIQKA-LRQRLGPEYISSRMAGG------GQKVCYIEGHRVINLANEM 78
RedB1                                                  40 DAQFIALLIVANQYGLNPWTKEIYAF-PDKQNGIVPV 76
RedB2  36                        KCDASDAQFIALLIVANQY-GLNPWTK-EIYAFPDKQN------GIVPVVGVDGWSRIINEN 89
RedB3   1  MSTALATLAGKLAERVGMDSVDPQELITTLRQTAFKGD-ASDAQFIALLIVANQYGLNP------WTKEIYAFPDKQNGIV-PV 76

               β3                     β4                   β5
        EEEEEEEEEEEEEEEEEEEEEEEEE  EEEEEEEEEEEEEE    EEEEEEEEEEEEEEEEEEEEE                   HHHHHHH

Rad52 79 FGYNG-WAHSI---T---QQN–VDFVDLNNGKFYVGVCAFVRVQLKDGS------YH-E-DVGYGVSEGLKS-----------KALSLEK 141
RedB1 77 VGVDG-WSRII---NENQQFDGMDFEQD------NESC-TCRIYRKDRN------HP-ICVTEWMDECRREP-----------FKTREGR 137
RedB2 90 QQFDG-MDFEQ---D---NES-----------------CTCRIYRKDRNHPI---CV-T-EWMDECRREPFKTREGREITGPWQSHPKRM 150
RedB3 77 VGVDG-WSRIINENQ---QFDGMDFEQDNES-------CTCRIYRKDR---NHPICV-T-EWMDECRREPF------------KT-REGR 137

             α3                                          α4                             α5
      HHHHHHHHHHHHHHHHHHHHHHHH                         HHHHHHHH                       HHHHHHH

Rad52 142 ------ARKEAV-TDG-LKRALRS----------FG-------NALG-N-CILDKDYLRS-LNKLPRQLPLEVDLTKAKRQDLEPSVEEARYNSCR  209
RedB1 138 ------EITGPWQSHP--KRMLR-----------HKAMIQCARLAFGFA-GIYDKDEAERIVEN                                 181
RedB2 151 ------LRHKAM-IQC-ARLAFGF----------AG-------I--Y-DKDEAERIVENT-AYTAERQPERDITPVNDETMQEINTLLIALDKTWD 217
RedB3 138 ------EIT-GP-WQSHPKRMLRHKAMIQCARLAFG--------FAG-I---YDKDEAER-IVENTAYTAERQPERDITPVNDETMQ         203
```

C

Figure S2. Model for the DNA binding domain of Redβ based on its homology with Rad52. (**A**) Model of Redβ (green) from Lopes *et al.* [2] superimposed on the structure of Rad52 (cyan) with two segments of ssDNA modeled as in Figure S1d. A significant extension and truncation in Redβ relative to Rad52 are indicated. (**B**) Model of Redβ as in panel A but with seven lysine residues labeled, to show their positions relative to the DNA. (**C**) Sequence alignments of three Redβ models with Rad52. RedB1 is from Erler *et al.* [3], RedB2 is from Lopes *et al.* [2], and RedB3 is from Matsubara *et al.* [4]. Lysine residues of Redβ are highlighted in blue with white lettering. Residues of Rad52 that contact the ssDNA at the inner site are highlighted in cyan, and at the outer site in green. Secondary structures of Rad52 are shown above the alignment.

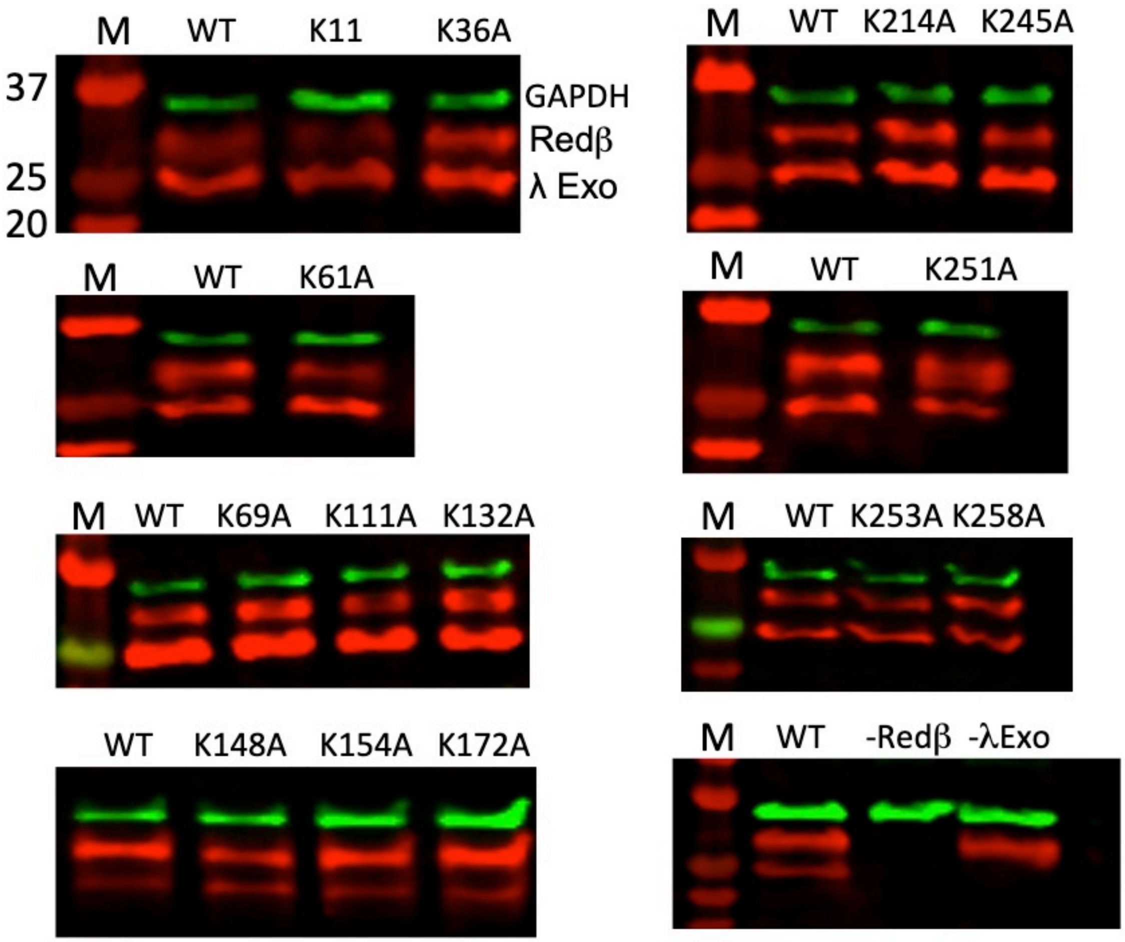

Figure S3. Western blots showing the levels of the 14 Lys to Ala Redβ variants expressed from their respective pSIM5 plasmids in HME57 cells. SDS-PAGE gels loaded with samples from the soluble fraction of each cell lysate were blotted on nitrocellulose and probed with anti-Redβ-λexo (anti-Redαβ; red color) and anti-GAPDH (green; loading control) antibodies as described in Materials and Methods. Lanes labeled "M" contain molecular weight standards (with sizes indicated in kDa at top left). The proteins corresponding to each band are indicated to the right of the gel at the top left. Expected sizes are 25.9 kDa for λ exo and 29.9 kDa for Redβ. A culture expressing WT Redβ and λ exo is included on each gel for reference. The bottom right panel shows controls for deletion mutants of Redβ and λ exo that contain stop codons inserted at codons 14 and 24, respectively [5,6].

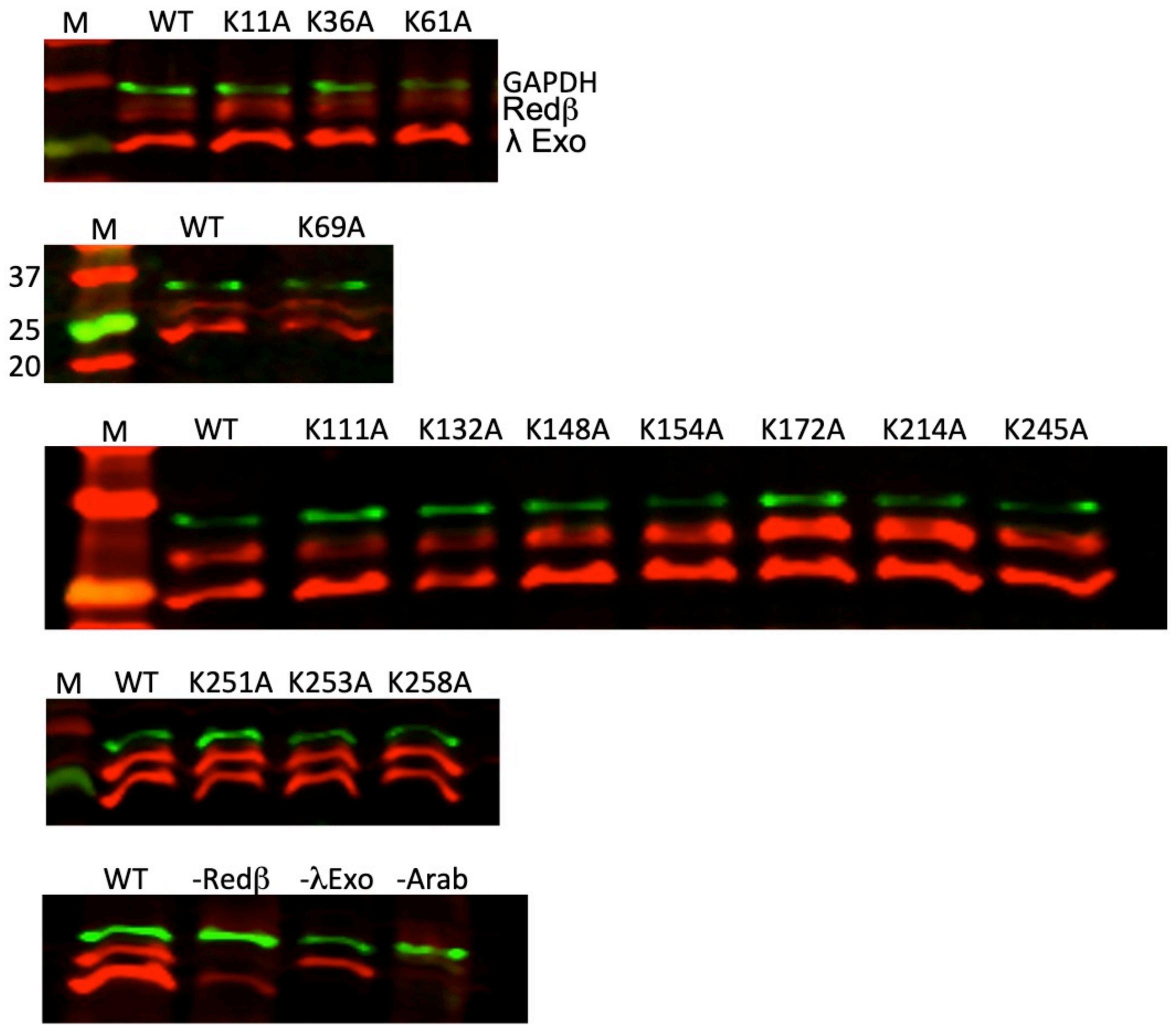

Figure S4. Western blots showing the levels of the 14 Lys to Ala Redβ variants expressed from the pSC101 plasmid in GB2005 cells. SDS-PAGE gels loaded with samples from soluble cell lysates for each variant were blotted on nitrocellulose and probed with anti-Redβ-λexo (anti-Redαβ; red color) and anti-GAPDH (green) antibodies as described in Materials and Methods. Lanes labeled "M" contain molecular weight standards (with sizes indicated in kDa at top left). The proteins corresponding to each band are indicated to the right of the gel at the top left. Expected sizes are 25.9 kDa for λ exo and 29.9 kDa for Redβ. A culture expressing WT Redβ and λ exo is included on each gel for reference. The bottom right panel shows controls for deletion mutants of Redβ and λ exo that contain stop codons inserted at codons 14 and 24, respectively [5,6], as well as a no-arabinose induction control.

Table S1. Activities of the 14 Lys to Ala variants in ssDNA recombination.[1]

	pSIM5			pSC101		
**Redβ Variant**	**Recomb. per 10^8 viable cells (x 10^5)**	**(*n*)**	**%WT**	**Recomb. per 10^8 viable cells (x 10^5)**	**(*n*)**	**%WT**
No DNA[2]	0	6	0	0	3	0
No Ind.[2]	0	6	0	0	6	0
WT	22 ± 31	40	100	2.1 ± 2.2	26	100
K11A	3.6 ± 3.8	9	17	0.29 ± 0.12	6	14
K36A	0.36 ± 0.55	8	1.7	0.055 ± 0.054	8	2.6
K61A	0 ± 0	9	0	0.00037 ± 0.00052	5	0.02
K69A	0.54 ± 0.41	9	2.5	0.059 ± 0.100	11	2.8
K111A	0.22 ± 0.11	8	1.0	0.029 ± 0.032	7	1.4
K132A	0.84 ± 0.83	9	3.8	0.0051 ± 0.0082	7	0.24
K148A	0.057 ± 0.059	8	0.26	0.027 ± 0.036	11	1.3
K154A	2.0 ± 2.5	9	9.2	0.00055 ± 0.00095	9	0.03
K172A	0.057 ± 0.089	9	0.26	0.0033 ± 0.0022	5	0.16
K214A	0.22 ± 0.37	8	0.99	0.0031 ± 0.0027	6	0.15
K245A	2.1 ± 4.5	8	9.5	0.062 ± 0.056	7	3.0
K251A	24 ± 21	7	110	0.94 ± 1.0	9	45
K253A	0.0069 ± 0.020	8	0.03	0.00070 ± 0.00046	6	0.03
K258A	4.6 ± 4.5	9	21	0.83 ± 0.82	6	40

[1]The data show the mean ± SD values for the number of recombinants observed for (*n*) independent experiments performed for each Redβ variant (WT or mutant). [2]The first two rows show negative controls where no repair oligonucleotide was added prior to electroporation (No DNA) or the cells were not induced for Red expression (No Ind.), either by 42°C for pSIM5 or by 0.2% arabinose for pSC101, prior to harvesting the cells at the normal time.

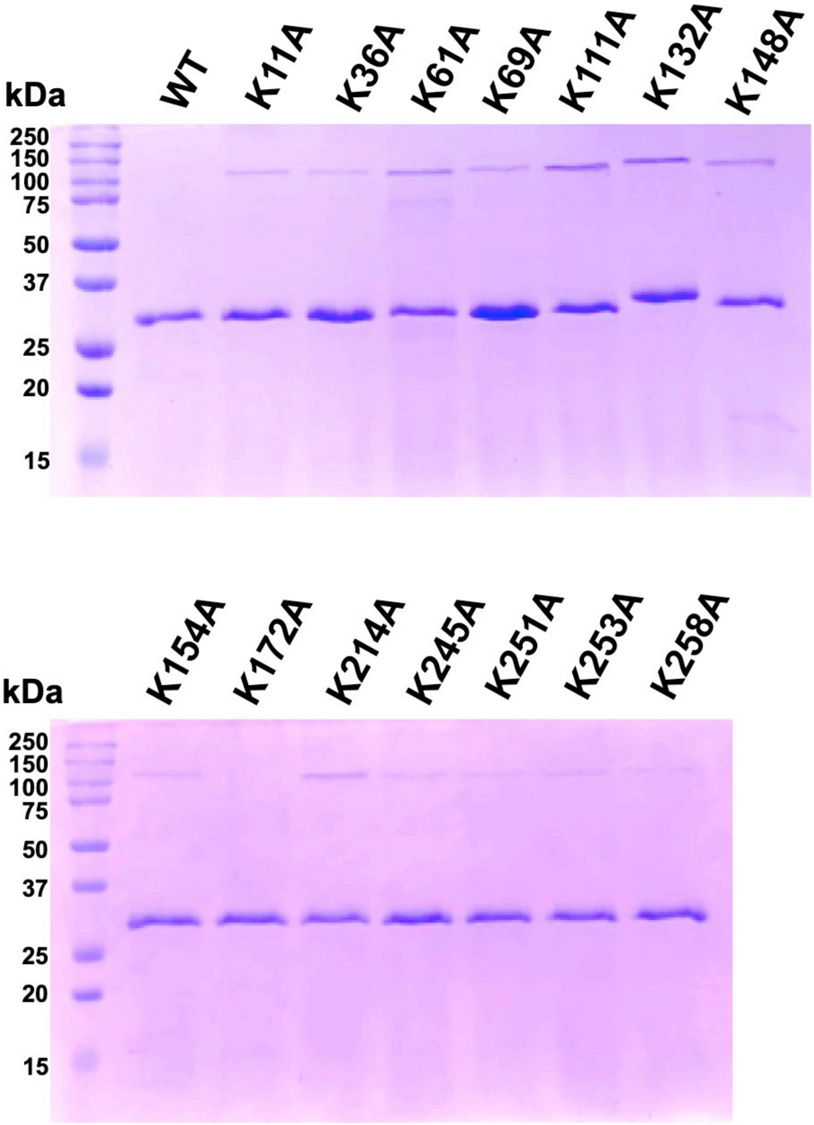

Figure S5. SDS-PAGE of purified protein for the 14 Lys to Ala Redβ variants. Each lane of the 12.5% SDS-PAGE gel was loaded with 1 μg of purified protein. For unknown reasons, the K132A protein runs anomalously high; the expression plasmid for this variant was double checked and confirmed to have the correct sequence for the expected 261 amino acid protein. The band at the top of the gel (between the 100 and 150 kDa markers) that is present in some of the samples is a non-denatured aggregate of Redβ that sometimes persists despite being heated to 95°C for 5 min in SDS loading buffer. Notice that this band is also higher for K132A, confirming that it is Redβ and not an impurity.

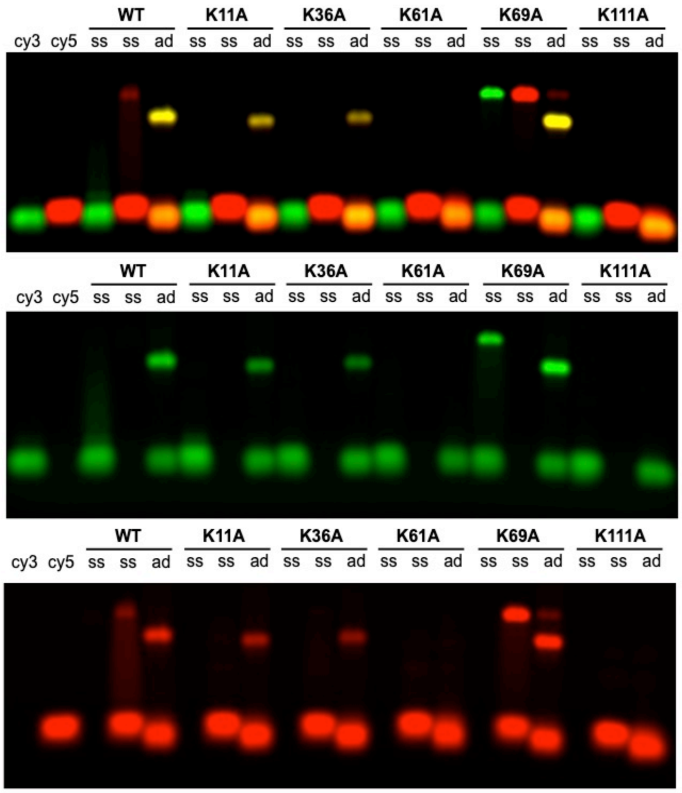
WT K11A K36A K61A K69A K111A
cy3 cy5 ss ss ad ss ss ad ss ss ad ss ss ad ss ss ad ss ss ad
WT K11A K36A K61A K69A K111A
cy3 cy5 ss ss ad ss ss ad ss ss ad ss ss ad ss ss ad ss ss ad
WT K11A K36A K61A K69A K111A
cy3 cy5 ss ss ad ss ss ad ss ss ad ss ss ad ss ss ad ss ss ad

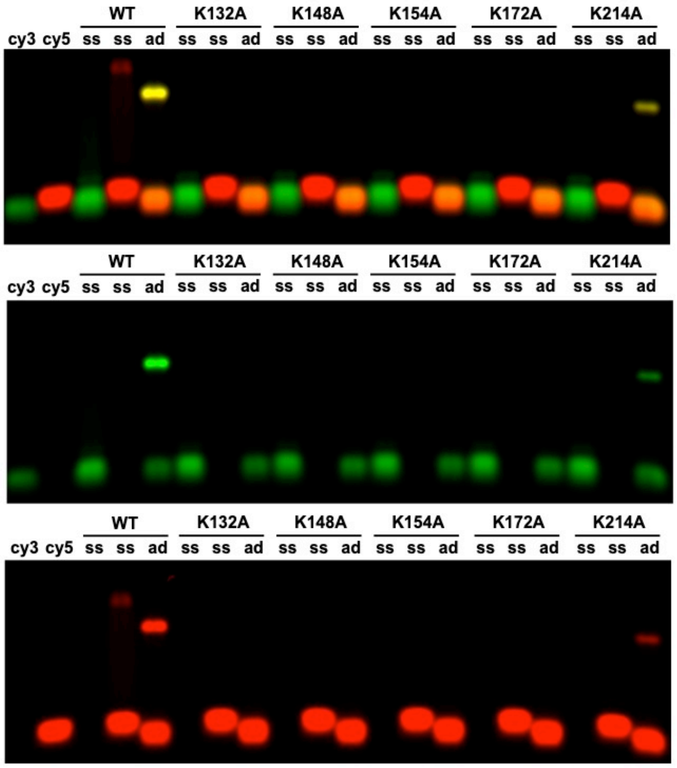
WT K132A K148A K154A K172A K214A
cy3 cy5 ss ss ad ss ss ad ss ss ad ss ss ad ss ss ad ss ss ad
WT K132A K148A K154A K172A K214A
cy3 cy5 ss ss ad ss ss ad ss ss ad ss ss ad ss ss ad ss ss ad
WT K132A K148A K154A K172A K214A
cy3 cy5 ss ss ad ss ss ad ss ss ad ss ss ad ss ss ad ss ss ad

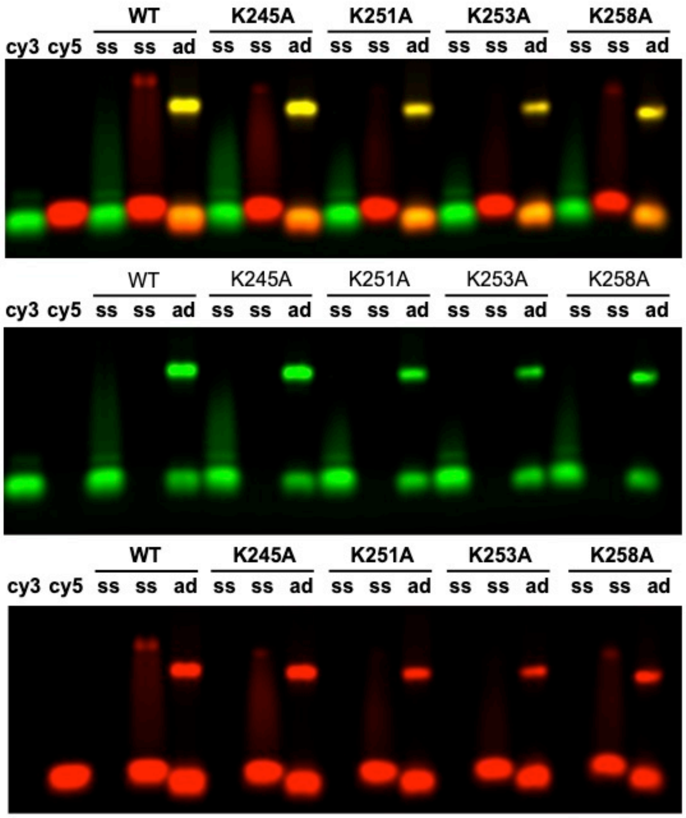

Figure S6. Gel-based annealing assays showing double and single-channel exposures. For each set of mutants, the top panel is a double channel exposure showing both the Cy3- and Cy5- (5'-) labeled 50mer oligonucleotides (taken from Figure 3), the middle panel is a single channel exposure showing just the Cy3-labeled 50mer, and the bottom panel is a single channel exposure showing just the Cy5-labeled 50mer. The single channel exposures confirm the presence or absence of each strand of ssDNA in each complex. Lanes labeled "cy3" and "cy5" contain single 50mer oligonucleotides without any protein added. Lanes labeled "ss" contain 10 μM Redβ mixed with 50 μM (nucleotides) of a single 50mer oligonucleotide. Lanes labeled "ad" contain Redβ mixed the two oligonucleotides added sequentially to form the complex with annealed duplex. Notice in lanes labeled "ad" that the yellow band for the complex with annealed duplex (when formed) contains both the Cy3 and Cy5 labeled 50mers.

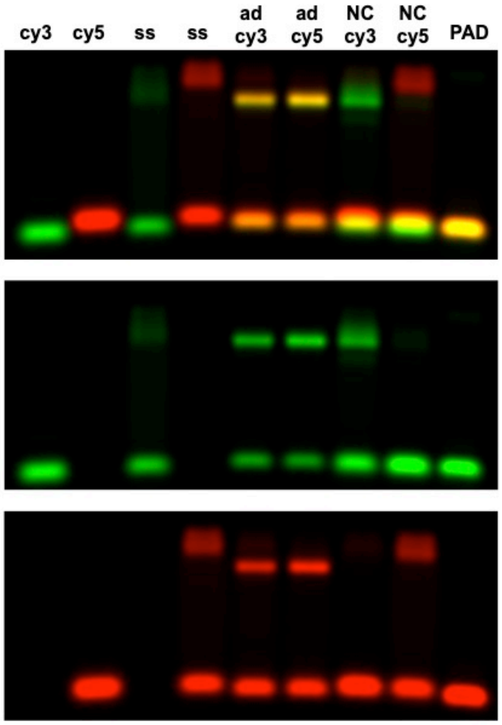

Oligonucleotides	Sequence
Cy5 50mer	5′-CCATCCGCAAAAATCGAGCTATGCAGGGCGATTCTGCTCTAAGCCATCCG-3′
Cy3 50mer	5′-GCGGATGGCTTAGAGCAGAATCGCCCTGCATAGCTCGATTTTTGCGGATG-3′
Cy3 NC 50mer	5′-GAGTGATAGGCATATGTGCTACGAGTATCGAAGGAACGGTCACTACGCAA-3′

Figure S7. Gel-based annealing assay for WT Redβ with additional control lanes. Lanes labeled “cy3” and “cy5” contain single 50mer oligonucleotides with no protein. Lanes labeled “ss” contain 50 μM (nucleotides) of one 50mer oligonucleotide (either Cy3 or Cy5) mixed with 10 μM of Redβ. Lanes labeled “ad” contain 10 μM of Redβ mixed with the two oligonucleotides added sequentially to form the complex with annealed duplex. For the lane labeled “ad Cy3” the “Cy3 50mer” was added first (and incubated for 15 min at 37°C), and then the “Cy5 50mer” was added (and incubated for an additional 15 min at 37°C). For the lane labeled “ad Cy5” the order of addition was reversed, with no impact on complex formation. For the lane labeled “NC cy3”, the “Cy3 NC 50mer”, which is non-complementary to “Cy5 50mer”, was mixed with Redβ, and then “Cy5 50mer” was added. This produced a complex with just the first DNA added. For the lane labeled “NC cy5”, the order of addition was reversed, and a complex with just the “Cy5 50mer” oligonucleotide was formed. For the lane labeled “PAD”, the two complementary oligonucleotides (Cy5 50mer and Cy3 50mer) were annealed to one another prior to addition of Redβ, to test for binding to pre-annealed duplex (no binding was observed). The top panel is a double channel exposure showing both oligonucleotides, while the next two panels show single-channel exposures for the Cy3 and Cy5-labeled 50mers, respectively. The bottom panel shows the sequences of each oligonucleotide.

Supplementary References (Zakharova *et al.*)

1. Saotome, M.; Saito, K.; Yasuda, T.; Ohtomo, H.; Sugiyama, S.; Nishimura, Y.; Kurumizaka, H.; Kagawa, W., 2018. Structural basis of homology-directed DNA repair mediated by RAD52. *iScience* **2018**, *3*, 50–62.
2. Lopes, A.; Amarir-Bouhram, J.; Faure, G.; Petit, M.-A.; Guerois, R. Detection of novel recombinases in bacteriophage genomes unveils Rad52, Rad51, and Gp2.5 remote homologs. *Nucleic Acids Res.* **2010**, *38*, 3952–3962.
3. Erler, A.; Wegmann, S.; Ellie-Caille, C.; Bradshaw, C.R.; Maresca, M.; Seidel, R.; Habermann, B.; Muller, D.J.; Stewart, A.F. Conformational adaptability of Redβ during DNA annealing and implications for its structural relationship with Rad52. *J. Mol. Biol.* **2009**, *391*, 586–598.
4. Matsubara, K.; Malay, A.D.; Curtis, F.A.; Sharples, G.J.; Heddle, J.G. Structural and functional characterization of the Redβ recombinase from bacteriophage λ. *PLoS ONE* **2013**, *8*, e78869.
5. Smith, C.E.; Bell, C.E. Domain structure of the Redβ single-strand annealing protein: The C-terminal domain is required for fine-tuning DNA-binding properties, interaction with the exonuclease partner, and recombination in vivo. *J. Mol. Biol.* **2016**, *428*, 561–578.
6. Pan, X.; Smith, C.E.; Zhang, J.; McCabe, K.A.; Fu, J.; Bell, C.E. A structure-activity analysis for probing the mechanism of processive double-stranded DNA digestion by λ exonuclease trimers. *Biochemistry* **2015**, *54*, 6139–6148.
